# Aerobic scope is sustained through a heatwave in juvenile Atlantic salmon (*Salmo salar*)

**DOI:** 10.1111/jfb.70347

**Published:** 2026-02-10

**Authors:** Lucy Cotgrove, Sergey Morozov, Miika Raitakivi, Evan Sala, Jenni M. Prokkola

**Affiliations:** ^1^ Migratory Fish and Regulated Rivers Natural Resources Institute Finland (Luke) Oulu Finland; ^2^ Organismal and Evolutionary Biology Research Programme, Faculty of Biological and Environmental Sciences University of Helsinki Helsinki Finland; ^3^ Infrastructure Unit Natural Resources Institute Finland (Luke) Laukaa Finland

**Keywords:** ecophysiology, energetics, respirometry, thermal performance

## Abstract

Aquatic ectotherms are vulnerable to heatwave‐induced physiological stress, which arises from increased energy demands and reduced dissolved oxygen content in warmer waters. Understanding thermal physiology is critical for predicting how commercially and ecologically important populations could be affected by the increasing risk of rising temperatures. Heatwave risk assessments often examine extremities of time scales: immediate impacts or long‐term consequences. However, little is known about how consistently increasing mid‐term thermal stress shapes aerobic performance in commercially important species such as Atlantic salmon (*Salmo salar*), which may face heat stress in rivers, especially at juvenile life stages. By measuring how salmon juveniles manage their aerobic capacity at 16, 19 and 22°C using intermittent flow respirometry, we test if their thermal performance curve declines at temperatures commonly occurring during heatwaves. Whole‐animal metabolism was measured from control individuals kept at 16°C before and after the heatwave, and after 4–5 days exposure at 19 and 22°C during the heatwave. We show standard metabolic rate increases with temperature, but maximum metabolic rate and aerobic scope do not change between these temperatures. These findings suggest that juvenile Atlantic salmon may have limited capacity to increase aerobic performance during moderate heatwaves, leaving them vulnerable to cumulative effects of oxygen limitation to vital functions such as growth and stress responses. As climate change intensifies, incorporating thermal performance curves into conservation strategies can be used for predicting population resilience and informing effective management.

## 1 INTRODUCTION

With climate change increasing the severity and frequency of heatwaves (IPCC, 2023), organisms may experience physiological thermal stress and even mortality due to extreme temperature fluctuations. Understanding organisms' thermal physiology is therefore critically important for conservation (Clark et al., 2013; Deutsch et al., 2008). Heatwaves frequently surpass the temperature range species have been adapted to, often cumulatively and repeatedly, imposing selection on thermal performance. This is especially true for ectotherms, whose internal temperatures are regulated by the surrounding environment, and in which most physiological processes including development, reproduction and metabolism are temperature‐dependent (Angilletta et al., 2010; Hochachka & Somero, 1973; Schulte, 2015; Seebacher, 2009). These processes can be described using thermal performance curves (TPCs), which illustrate how biological rates, such as oxygen consumption or activity per unit time, change with temperature (Schulte et al., 2011).

TPCs can be estimated using thermal windows, critical temperatures and preferred temperature ranges (Jørgensen et al., 2021; Ørsted et al., 2022; Peralta‐Maraver & Rezende, 2021). They can be used to compare inter‐ and intraspecies responses to anticipated temperatures under future climates and how these may vary across life stages (Lefevre et al., 2021; Seebacher & Little, 2021). In water‐breathing aquatic ectotherms, such as fishes, TPCs linked to demand, uptake and delivery of oxygen are particularly important because of the decreasing solubility but increasing oxygen consumption in warmer water (Little et al., 2020; Little & Seebacher, 2021; Seebacher & Little, 2021; Verberk et al., 2022). Consequently, TPCs related to metabolic rates and aerobic performance are pivotal to understanding effects of heatwaves on fishes.

Metabolic rate is a fundamental aspect of fish physiology, reflecting energy expenditure associated with basic life processes such as respiration, digestion and movement (Brett, 1964; Fry et al., 1947). Standard metabolic rate (SMR) of an animal represents the baseline rate of aerobic metabolism required to sustain life in a post‐absorptive, resting state, whereas the maximum capacity for aerobic performance is set by maximum metabolic rate (MMR) (Sandblom et al., 2016). The difference between SMR and MMR represents aerobic scope (AS) of an animal and theoretically determines the capacity of aerobic metabolism to support key life‐history attributes such as activity, growth and reproduction, each of which has a specific oxygen cost (Fry, 1971; Fry et al., 1947). Therefore, reductions in AS can impair physical performance (Johansen & Jones, 2011; Pörtner & Farrell, 2008; Priede, 1985). Given SMR of ectotherms rises with temperature and often no such increase is detected in MMR (Fry et al., 1947; Norin et al., 2014; Sandblom et al., 2016), increases in temperature beyond optimum can result in a decline in AS (Farrell, 2016; Fry, 1971; Lefevre et al., 2021). However, maintaining growth and other aerobic functions with increasing temperature requires an increasing AS, because of the higher costs of activities such as digestion and assimilation and swimming (Jahn & Seebacher, 2022; Jutfelt et al., 2021). Therefore, not only a declining A, but also a plateauing AS may cause declining growth, health or survival of individuals (Alfonso et al., 2021; Jutfelt et al., 2021; Navarro et al., 2019; Sadoul & Vijayan, 2016). Physiologically, a plateau in AS at higher temperatures suggests oxygen supply can limit performance as temperature increases (Christensen et al., 2021; Hvas et al., 2017; Závorka et al., 2020). Both declines and plateaus of AS pose significant risks for salmonids, which include economically and culturally important, cold‐water adapted species. In these fish, limitations of AS have already been linked to reduced growth, survival or collapse of aerobic capability during heatwaves (Hvas et al., 2017; Eliason et al., 2013; Pörtner & Farrell, 2008; Wade et al., 2019).

The Atlantic salmon (*Salmo salar* L. 1758) is faced with supra‐optimal temperatures especially at riverine life stages, including juveniles (parr) and adults during their spawning migration, with reports of present‐day river temperatures up to 28°C (O'Sullivan et al., 2023; Strøm et al., 2019). Juvenile *S. salar* rear for 1–5 years in fresh water before migrating to the sea (Aas et al., 2010), with potential to encounter multiple heatwaves. These can have direct effects on survival, but also cascading effects on population dynamics through reduced growth rates. Faster freshwater growth in salmonids is linked to higher survival and faster maturation at sea (Hutchings & Jones, 1998; Simpson, 1992; Thorpe, 2007). In some populations, a negative association between freshwater growth and river temperature has been detected (Alioravainen et al., 2023). For the majority of their life cycle at sea, *S. salar* will experience temperatures below 8°C (review by Strøm et al., 2020; Jensen et al., 2014; Lacroix, 2013; Reddin, 1985). They tend to avoid temperatures above 15°C (Fisher & Elson, 1950; Johansson et al., 2009; Lacroix, 2013), and their optimal temperature range for feeding and normal behaviour in the wild has been suggested to be 6–20°C, with peak growth rates occurring at 16–17°C in aquaculture conditions (Dwyer & Piper, 1987; Elliott, 1982). Previous work assessing thermal performance in *S. salar* has often focused on upper limits during acute exposure of less than 24 h (Desforges et al., 2023 and papers within), or long‐term stable temperature increases over several months (Anttila et al., 2014; Del Rio et al., 2021; Hvas et al., 2017). When AS has been measured in a heatwave context in a related species, Casselman et al. (2012) found AS peaked at 17°C, and decreased until testing finished at 21°C in juvenile Coho salmon (*Oncorhynchus kisutch* Walbaum 1792). In post‐smolt *S. salar*, AS tended to increase (a non‐significant effect) as temperature increased from 13 to 23°C, but swimming ability and feeding rate greatly decreased and mortality increased, when metabolic rates of *S. salar* were tested in groups without a cumulative exposure (Hvas et al., 2017). Further, thermal variability in acclimation regime had no effect on *S. salar* AS compared to a stable acclimation when metabolic rates were tested acutely at the same temperature (Morissette et al., 2021). Still, information on cumulative effects of thermal fluctuations over several days or weeks, which better reflect natural heatwave patterns, is limited (Morash et al., 2021; Nuic et al., 2024). This information is pivotal for conservation of *S. salar*. The decline in salmon populations has already reduced opportunities for commercial and recreational fishery, cultural traditions and increased risk of local extinctions (Dadswell et al., 2022; ICES, 2024a, 2024b).

Here, we test how mid‐term increasing temperatures that are prevalent in rivers during heatwaves impact metabolic rates and AS in hatchery‐reared juvenile *S. salar*. We hypothesize SMR will increase as temperature rises. We expect MMR to show little variation between temperatures, and therefore a decrease in AS as temperature increases. These findings will provide insight into physiological constraints juvenile *S. salar* may face during heatwaves, using an ecologically relevant temperature regime.

## 2 METHODS

### 2.1 Fish husbandry

The experiment was performed with a permit granted by the Finnish Project Authorisation Board (no. ESAVI/16748/2023). Families of *S. salar* were bred using a hatchery broodstock originating from River Iijoki, maintained by Natural Resources Institute of Finland (Luke), in Taivalkoski, Finland. The broodstock was established in 2012 from parents that were originally stocked into the river as juveniles/smolts, and therefore completed half of their life cycle in the wild. Individuals from the broodstock were crossed to make full‐sib families of offspring in October 2022. Embryos were incubated in Taivalkoski hatchery in family‐specific flow‐through trays at natural temperatures of incoming water from River Ohtaoja, and dead embryos were removed regularly.

**FIGURE 1:**
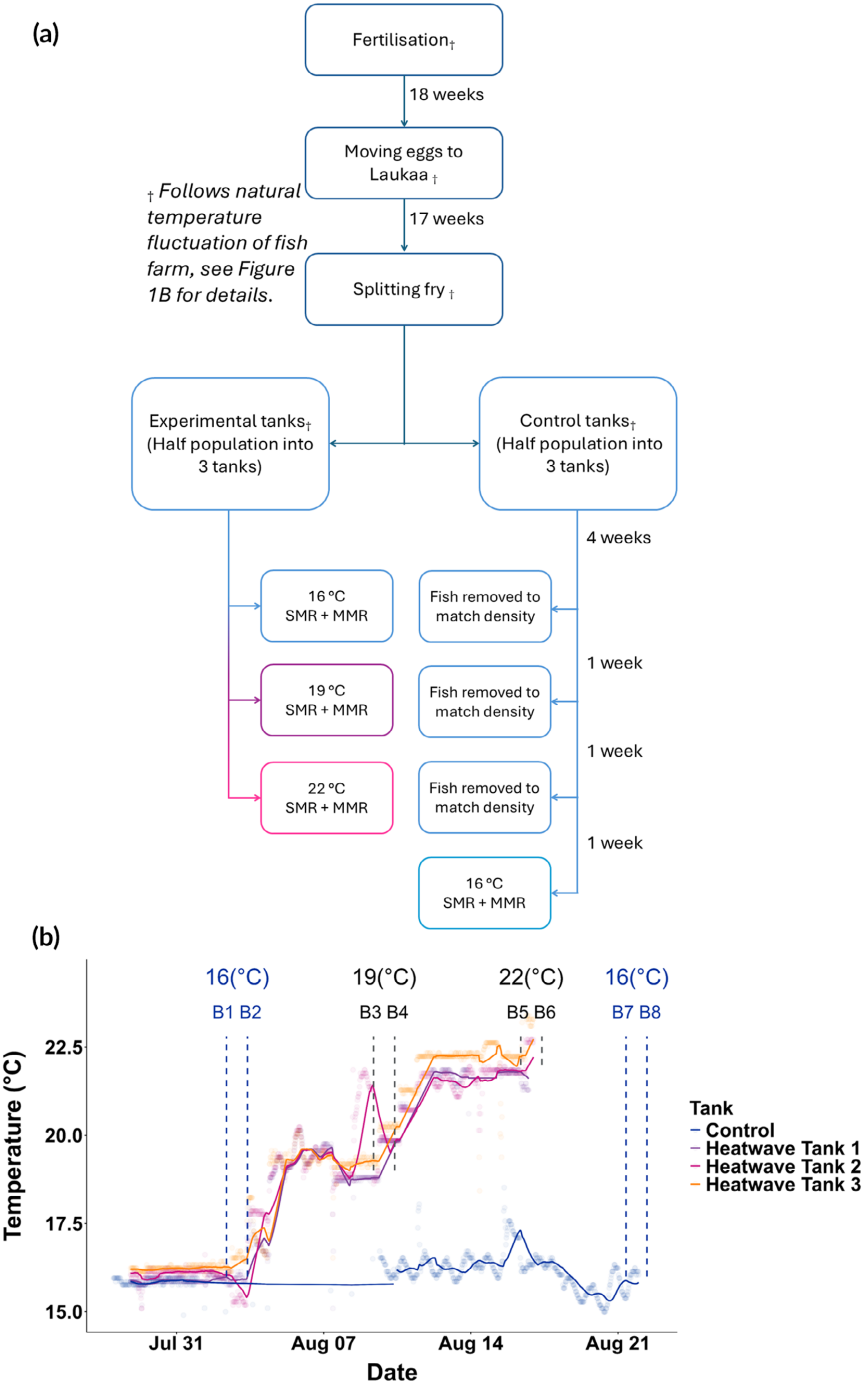
(a) Outline of heatwave experiment with acclimation times for juvenile *Salmo salar*; (b) temperature regime *S. salar* experienced. Solid lines and points represent temperatures (mean of 16, 19 and 22°C) of the heatwave tanks (three tanks with independent temperature regulation) and control tanks (three tanks with an identical temperature, 16°C) during the experiment. Lines fitted using a 40‐point rolling average, where each point is 1 h. Dashed vertical lines (B1–B8) indicate timing of metabolic rate measurements: Two batches measured each week. Fish were removed from heatwave tanks into H‐Tronic controlled acclimation tank 24 h before testing. Variation in temperature profiles at each step is related to natural mixing in the incoming lake water.

Embryos were disinfected by iodine bath (Buffodine) on 9 March 2023, and transported to a LUKE hatchery in Laukaa, Finland, where experiments were conducted (Figure 1a). Survival between transportation and start of the experiment was 22%. Excess mortality may have been caused by the closeness to the embryo's hatching date and transporting to the fish farm (no disease was observed in the offspring). Embryos from three unrelated families were incubated and reared in separate circular tanks (diameter 80 cm) and supplied with a continuous flow of filtered water from a mix of local lake Peurunkajärvi and river Peurunkajoki. Feeding of alevins was started with powdered feed (BioMar Group) when most of the egg yolk was consumed approximately on 25 May 2023. Tanks were cleaned by scrubbing surfaces and siphoning excess food twice a week until 10 June 2023, and then daily until the end of the experiment. Feed rations were calculated from growth predictions assuming feed conversion efficiency of 0.8 (Elliott & Hurley, 1997), and adjusted such that a small amount of feed was left uneaten. Fish were fed using 12 h belt feeders with the daily ration, except for 1 day preceding metabolic measurements, when fish were fasted. During summer and the experiment, fish were reared under constant light. Rearing temperature raised gradually with natural water temperatures over approximately 3 months until the experiment was started; temperature varied from 9.2 to 15.7°C between first feeding and experimental period.

At the start of the experiment 27 July 2023, families were divided into two groups, control and heatwave, with approximately same number of individuals in each group within a family (*N* = 24–62 within family per tank). Fish were kept in three tanks each for control and heatwave groups. Densities within families were kept similar between control and heatwave tanks throughout the experiment by simultaneously removing fish from control tanks to match densities in heatwave tanks (Figure 1a). Control tanks were kept at close to 16°C (mean = 16.1, SD = 0.5) throughout the experimental period by regulating incoming water flows from the river and the lake with a natural temperature difference (Figure 1b). Temperatures in one control tank (the same water source used in all three tanks) and the three heatwave tanks were measured every 30 min with Hobo 64 K Pendant temperature loggers (Hobo, Bourne, MA, USA).

Temperatures of the heatwave tanks were regulated via a heating tank to offset colder temperatures of the room and incoming water using a temperature controller (TS1000, H‐Tronic GmbH, Germany) with a temperature probe PT‐1000 located in each tank, and an Eheim 600 or 300 pump (Eheim, Deizisau, Germany) depending on distance from the heating tank. Pumps circulated water through stainless steel coils in the heating tank, in which temperature fluctuated between 30 and 35°C. Heatwave tank water was aerated constantly to prevent declines in dissolved O_2_ related to increasing temperatures, as the flow‐through rate of fresh water to the tanks was reduced (approx. 1 L min^−1^) during the heatwave to maintain temperatures.

### 2.2 Experimental heatwave

Fish in the heatwave treatment were exposed to three test temperatures: 16, 19 and 22°C (Figure 1a,b). First, 16°C was maintained as a control temperature for 8 days, after which 3–8 individuals from each family were randomly picked for measurements of metabolic rate (similar sample size across temperatures within a family), and moved into a fasting tank, where they were unfed until the measurements started the following day. Temperature of the fasting tank was the same as heatwave tanks. After the measurement of MMR and SMR at 16°C, temperature was increased by 1° per day for 3 days, and maintained for 4–5 days, after which metabolic rate measurements were taken from a new set of fish at 19°C. This was repeated, and metabolic rate measurements were taken at 22°C. Due to a malfunctioning temperature controller at 19°C, there was an increase in temperature in one of the heatwave tanks to 21.5°C (Figure 1b), but there was no significant difference in family or between batches tested at the same temperature post exposure. Metabolic rate experiments were conducted once per individual, and fish were killed after SMR measurements.

To measure MMR, fish were transferred from the fasting tank into 50 L buckets filled with 5 cm of water at the testing temperature using plastic cups of water to reduce air exposure. Fish were encouraged to swim by hand around the circular container (Prokkola et al., 2022; Raby et al., 2020). Chasing lasted 2 min, and all fish were unresponsive to the touch of caudal fins, thus determined fatigued. Fish were then rapidly transferred to respirometry chambers using plastic cups with water. Two people simultaneously chased one fish each, and each batch of up to 16 individuals was processed within 2 h. Fish were left in respirometry chambers overnight to capture MMR and SMR for each fish, as per Chabot, McKenzie, & Craig (2016) and Chabot, Steffensen, & Farrell (2016). Chambers were flushed with fresh water from the tank for 5 min every 15 min, allowing fully aerated water to enter the chamber. The lowest recorded oxygen level in any chamber was 6.7 mg L ^−1^ at 22°C. After SMR measurements, fish were killed using an overdose of MS‐222, measured and weighed.

Immediately before and after metabolic measurements, background respiration was measured in empty chambers for three measurement cycles (15 min measurement, 5 min flush), and an average of these slopes was used to adjust data for MMR and SMR. For those trials conducted immediately after the previous one, post‐trial background data of the previous trial were used as pre‐trial background values. To prevent bacterial build‐up, the system was bleached between temperature changes, and all chamber parts scrubbed to limit bacterial growth and rinsed thoroughly. Additionally, water in the respirometry tank was continuously circulated through a UV filter.

A total of 120 fish were subjected to intermittent flow respirometry after a 24 h fasting period to provide estimates of metabolic rate (SMR, MMR, AS; see Table S1) (Killen et al., 2021; Svendsen et al., 2016). Due to death in respirometer chambers, exclusion due to outliers, incomplete data recording and due to computer failure, 72 fish were included in the analysis (Table S2).

### 2.3 Respirometer design

A summary of the respirometry design and measurement protocol is provided in Table S1, following Killen et al. (2021). A 16‐chamber intermittent flow respirometer, each chamber containing an individual fish, was submerged in a temperature‐regulated water bath (16, 19 and 22 ± 0.1°C; 200 L) that was saturated with air. The oxygen content of water in chambers was measured every 2 s using a four‐channel fibre optic oxygen meter with associated oxygen sensors and software (FireStingO2; PyroScience GmbH, Aachen, Germany). Before the first measurements at each temperature, all 16 O_2_ sensors were calibrated simultaneously for 0% oxygen saturation with O_2_‐free water, made using sodium sulphate. Immediately after, the 16 sensors were simultaneously calibrated for 100% oxygen saturation with air‐saturated water prepared using an air stone. Temperature‐compensation for O_2_ saturation was based on PT100 temperature sensors, which were placed in the middle of the respirometer tank and connected to each of the Firesting meters. Chambers were shielded from disturbance and light using an opaque plastic cover during the measurement of SMR. Flush and recirculation pumps were controlled using PumpResp controllers (4‐channel model, FishResp, Finland, https://github.com/embedded-sergey/PumpResp, [Morozov, 2024]). The temperature of the respirometer tank was maintained using a temperature controller (as in the heatwave tanks) and a reservoir connected to a TECO 2000 Chiller/heater. The reservoir received constant inflow of water (approx. 1 L min^−1^) from the same source as rearing tanks to maintain good water quality. Chambers for the respirometer were made using glass tubing (120 mm length, inner diameter 38 mm, wall thickness 3.2 mm) and plastic caps that were 3D‐printed on PA2200 polyamide. ‘HeiBer’‐caps were designed by Heidrikur Bergsson, University of Copenhagen (https://zenodo.org/record/4062429#.YMSW7h1RVTY). Volume of chambers and tubing was 131.86 ± 1 mL (the exact volumes per chamber are reported in an online data repository, see Data Availability). Water inside chambers was mixed by a plastic disk attached to each cap to distribute flow. Disks were 3D printed using the same method as the respirometry caps and attached with stainless steel screws. Caps were sealed using rubber O‐rings and connected to the flush and recirculation pumps and valves with gas‐impermeable Tygon tubing (Tygon S3 E‐3603, Saint‐Gobain, Paris, France). Recirculation system was confirmed to be waterproof by filling with water and plugging the flush pump and probe connections. Water was recirculated using one submersible pump (12 V DC, 6 W 2 L min^−1^ pump, Qingdao Xinhui Hardware Machinery Co., Ltd., Qingdao) per channel within the recirculation loop. Chambers were flushed using a second pump (same as previous), using an inflow of water from the tank into the chambers, and excess water was flushed through the chamber to a flush pipe which was placed with the end above the water surface. This allowed fully aerated water to enter the chamber. Flow rate through both flush line and recirculation loop could be controlled by valves attached within the flow. Flow rate of the respirometers was approximately 0.1 (±0.008) L min^−1^ without fish. Information on time and pump phase (either flush or measurement) was recorded by the PumpResp controllers and stored on a computer. Flow had no apparent effect on movement of fish within chambers in SMR or MMR measurements.

## 3 DATA ANALYSIS

All data and statistical analyses were done in R environment v.2024.04.2 (R Core Team, 2024). FishResp package (Morozov et al., 2019) was used to filter and calculate metabolic rate estimates. Slopes of oxygen consumption were adjusted for bacterial oxygen consumption using pre‐and post‐background measurements, assuming a linear change. For SMR, oxygen consumption (mg O_2_ h^−1^) for each measurement phase was derived from the slope of linear regression of dissolved oxygen concentration over time. SMR slopes were quality filtered and smoothed before analysis to exclude non‐linear declines of oxygen: first, slopes were filtered based on *R* ^2^ > 0.95, then slopes that did not meet the *R* ^2^ criteria were smoothed using a running mean of 29 s (Chabot et al., 2021), then *R* ^2^ filter was re‐applied and, finally, linearity was checked visually from all slopes that met the criteria of *R* ^2^ > 0.95. The first and last 60 s of measurement periods were excluded to account for mixing of water and changes in flow due to flushing. Mean of the lowest normal distribution (MLND) was used to estimate SMR for each individual from extracted slopes (Chabot, McKenzie, & Craig, 2016); Chabot, Steffensen, & Farrell, 2016). Slopes for MMR were extracted using a derivative of a polynomial curve fitted on each measurement (function *smooth.spline*, df = 10) as in the *spline‐MMR* method (Prokkola et al., 2022), that is, from the beginning of the MMR measurement, when respiration was highest. Slopes were then used to calculate MMR (mg O_2_ h^−1^) using *FishResp*. AS was calculated as the difference between absolute MMR and SMR.

**FIGURE 2:**
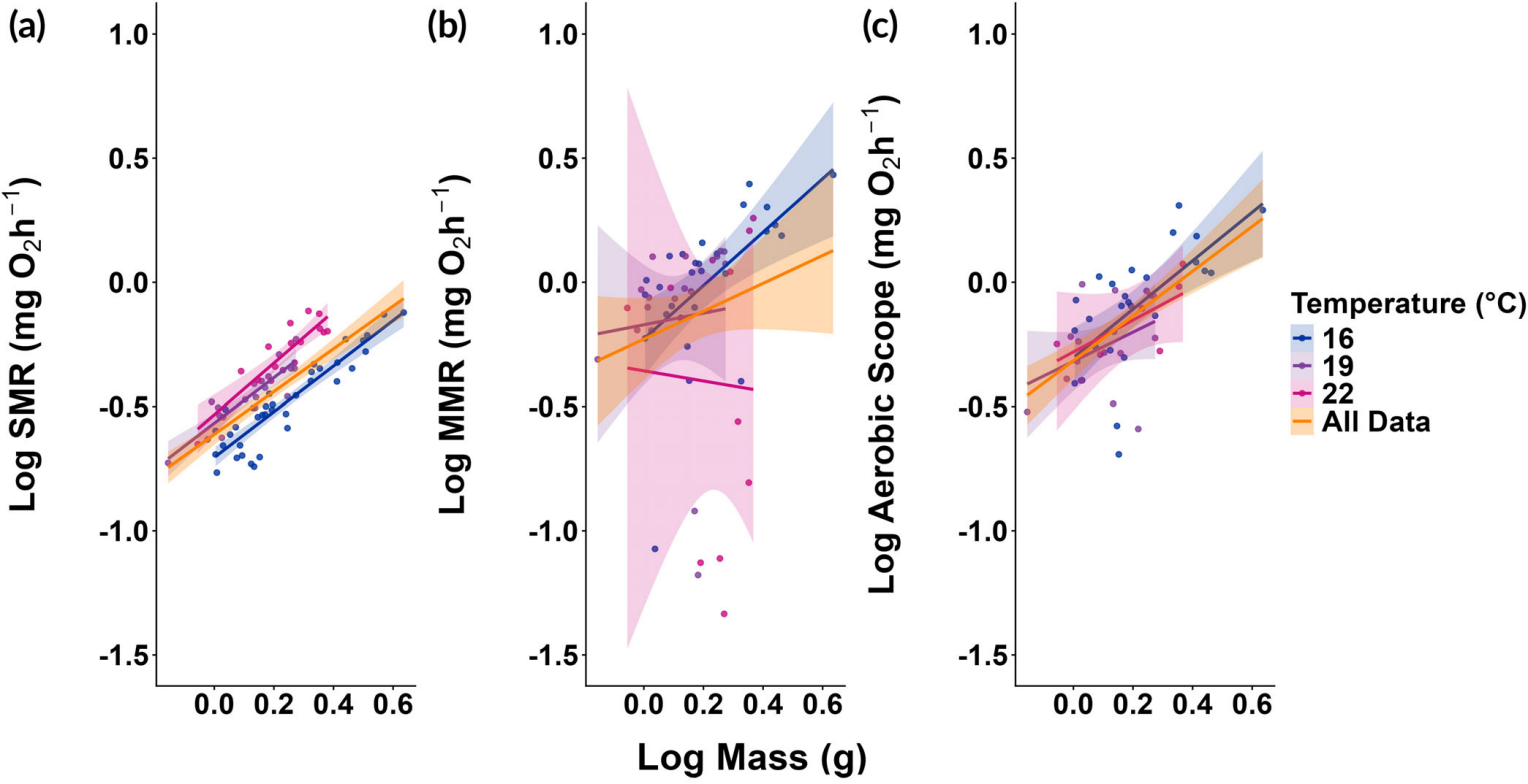
Scatter plots to show the relationship of log‐transformed metabolic rates with log‐transformed mass in juvenile *Salmo salar* [a: standard metabolic rate (SMR), b: maximum metabolic rate (MMR), c: aerobic scope (AS)]. Each point represents individual fish, with different colours representing different tested temperatures (16°C: blue, 19°C: purple, 22°C: pink). Goodness‐of‐fit lines indicate linear regression and shading shows 95% confidence intervals for each temperature, with orange colour representing fit of all data.

A linear mixed‐effects model was fitted (estimated using restricted maximum likelihood and *nloptwrap* optimizer) to predict SMR and MMR, and a non‐linear mixed‐effects model was fitted to predict AS, all of which were log transformed. Homoscedasticity and normality of residuals were assessed by visual inspection of residual plots, and if residuals were not normal, a non‐linear model was used. Temperature and log‐transformed mass were included as fixed effects (formula: Metabolic Measurement ~ Temperature + Mass). The model included batch as a random effect (formula:~1|Batch) (Zuur et al., 2009). 95% Confidence intervals (CIs) and *p*‐values were computed using a Wald t‐distribution approximation. Post hoc comparisons of temperatures were performed using Tukey method of comparing estimates, and *p*‐value <0.05 was considered significant. Statistical models were fitted using *nlme*, *lme4* and *lmerTest* packages in R, and post hoc analysis was performed in *emmeans* package (Bates et al., 2015; Kuznetsova et al., 2017; Lenth, 2025; Pinheiro et al., 2025). For visual representation, SMR, MMR and AS data were mass‐adjusted to account for hypo‐allometric scaling of metabolic rates by linear regression of log_10_‐transformed metabolic rates against log_10_‐transformed body mass (Figure 2). Finally, Pearson's correlations were calculated among either absolute or mass‐adjusted metabolic variables.

## 4 RESULTS

In total, data of 72 fish were used at three temperatures across four consecutive weeks: 16, 19 and 22°C and 16°C again (Tables 1 and S3). Metabolic rates and AS scaled positively with body mass, with scaling exponents from 0.86 to 1.1 (Figure 2; Tables 2 and S4).

**TABLE 1:** Descriptive statistics for mass (g) and length (mm) for juvenile *Salmo salar* tested at different temperatures.

		Mass (g)				Length (mm)			
Temperature (°C)	*n*	$\bar{x}$	σ	Min	Max	$\bar{x}$	σ	Min	Max
16 (week 1)	22	1.42	0.41	1.01	2.59	57.64	5.38	50	69
19	19	1.35	0.35	0.70	1.88	56.84	4.71	47	64
22	16	1.79	0.42	0.88	2.40	61.00	4.35	54	68
16 (week 4)	15	2.56	0.88	1.40	4.32	68.53	8.22	56	82
Total	72	1.72	0.70	0.70	4.32	60.44	7.16	47	82

Abbreviations: Max, maximum value; Min, minimum value; *n*, number of individuals; x̄, mean metabolic rate; σ, standard deviation.

**TABLE 2:** Model summaries for metabolic rate estimates for juvenile *Salmo salar*, including fish body mass and temperature (16 vs. 19 and 22°C) as independent variables.

	log10 (SMR)			log10 (MMR)			log10 (AS)		
	Est.	CI	*p*	Est.	CI	*p*	Est.	CI	*p*
log10 (mass)	**0.922**	**0.057**	**<0.001**	**0.978**	**0.120**	**<0.001**	**1.044**	**0.229**	**<0.001**
19°C	**0.131**	**0.027**	**0.004**	0.011	0.046	0.826	−0.038	0.086	0.674
22°C	**0.198**	**0.027**	**<0.001**	0.040	0.046	0.424	−0.033	0.086	0.717
Intercept	**−0.704**	**0.021**	**<0.001**	**−0.152**	**0.038**	**0.004**	**−0.342**	**0.072**	**<0.001**
*Random effects*									
Intercept SD (Batch)	0.0215			0.0116			0.0116		
Residual SD	0.0638			0.1492			0.1492		
Marginal *R* ^2^	0.849			0.521			0.521		
Conditional *R* ^2^	0.864			0.524			0.524		

*Note*: Batch is included as random effect. Est. indicates estimate. CI indicates 95% confidence intervals; *p* indicates significance, with bolded results showing *p* < 0.05.

Abbreviations: AS, aerobic scope; MMR, maximum metabolic rate; SMR, standard metabolic rate.

**FIGURE 3:**
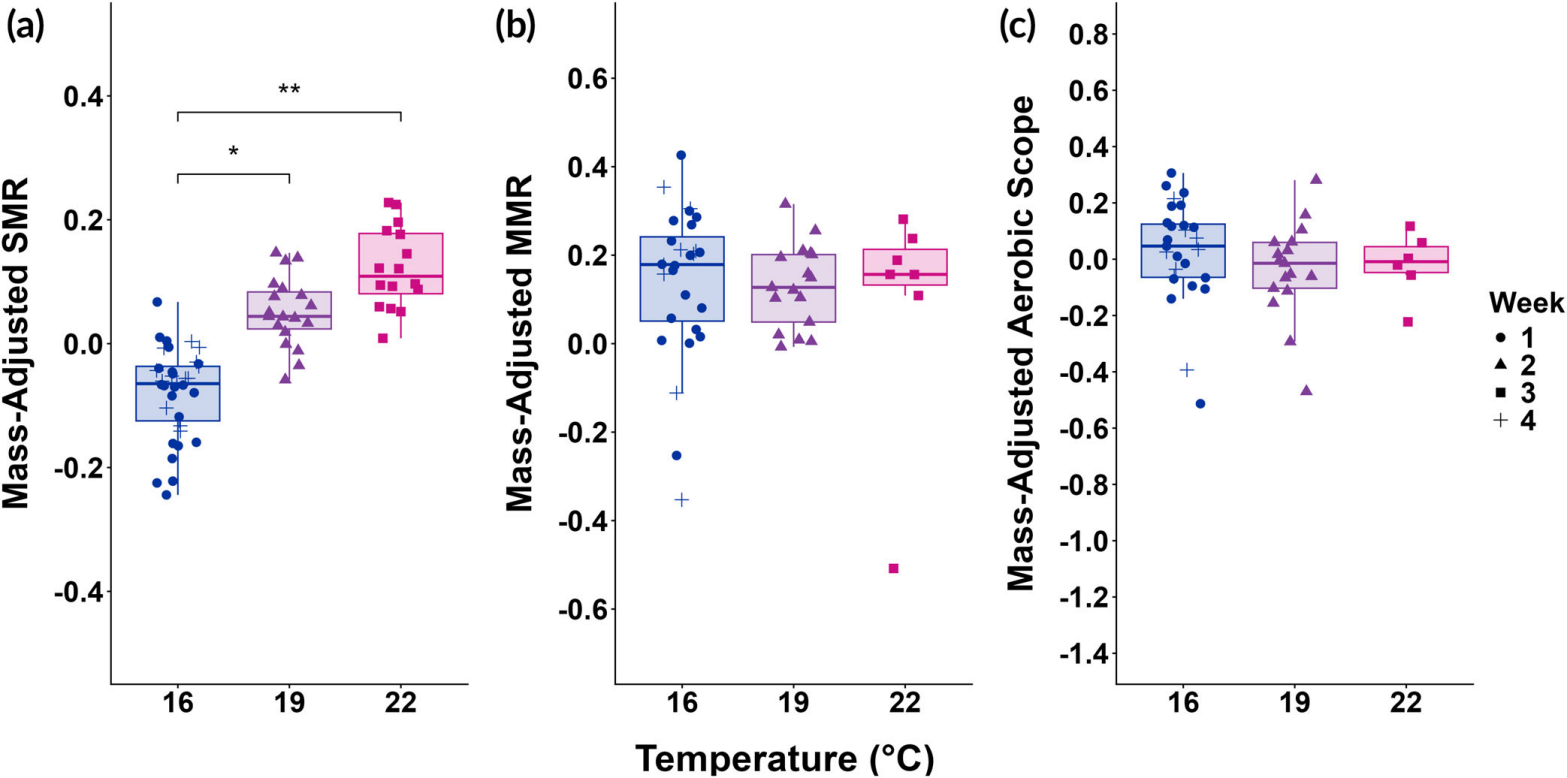
Boxplots to show the relationship between temperature and mass‐adjusted metabolic rate measurements: (a) standard metabolic rate (SMR), (b) maximum metabolic rate (MMR) and (c) aerobic scope (AS) in juvenile *Salmo salar*. Different point shapes indicate different weeks of measurement, and different colours indicate different temperatures. Asterisk and brackets show significant differences (*p < 0.05 and **p < 0.01).

We found temperature to be a significant predictor of SMR (16–19°C: *t*(66) = 4.80, *p* < 0.001; 16–22°C: *t*(66) = 7.43, *p* < 0.001, Table 3). SMR was significantly higher in 19 and 22°C than in 16°C, but there was no difference between 19 and 22°C (Figure 3, Table 4). There was a 57% increase in predicted SMR for 6°C increase in temperature (Table 4). However, there was no significant change in MMR or AS between temperatures (Figure 3; Table 2).

**TABLE 3:** Post hoc ANOVA contrasts from the results of the SMR model described in Table 2, comparing the temperature treatments 16, 19 and 22°C.

Comparison	Estimate	SE	df	*t*	*P*
16–19°C	−0.13	0.03	4.66	−4.71	**0.014**
16–22°C	−0.20	0.03	4.73	−7.42	**0.002**
19–22°C	−0.07	0.03	4.94	−2.13	0.178

*Note*: SE shows standard error, df degrees of freedom and the associated *t* ratio and *p* indicate significance, with bolded results showing *p* < 0.05.

Abbreviation: SMR, standard metabolic rate.

**TABLE 4:** Table showing predicted SMR (mg O_2_ h^−1^) and 95% confidence intervals (CI) for a mean mass juvenile *Salmo salar* (1.72 g) at different temperatures.

Temperature (°C)	Predicted SMR (for a 1.72 g fish)	Lower CI	Upper CI
16	0.33	0.24	0.44
19	0.44	0.32	0.58
22	0.52	0.39	0.70

Abbreviation: SMR, standard metabolic rate.

### 4.1 Correlations among metabolic variables

At all temperatures, MMR and AS were highly positively correlated (*r* > 0.9) in both raw and mass adjusted data. Absolute SMR and MMR were positively correlated at 16 and 22°C. Absolute SMR and AS were positively correlated at 16°C. However, mass adjusted SMR and AS were negatively correlated at 19 and 22°C (Table S1, Figure S1).

## 5 DISCUSSION

This study demonstrates that rising temperatures lead to an increase in SMR in juvenile *S. salar*. However, there was no change in MMR or in AS across tested temperatures. Although an increase in SMR with temperature is well established in ectotherms due to effects of temperature on biochemical rates (Clark et al., 2013; McKenzie et al., 2021; Raby et al., 2016), there are more mixed results on the relationship of MMR, AS and increasing temperatures in fishes. Although AS is defined as the difference between MMR and SMR, we did not detect a significant reduction in AS, even though SMR significantly increased and MMR plateaued at higher temperatures. This apparent inconsistency likely reflects variability in MMR, which is inherited by the AS calculation and adds noise to data. While non‐significant, MMR slightly increased with temperature, and AS generally declined. Correlations among variables also varied across temperatures, though MMR and AS were consistently positively correlated. Together, these factors contribute to the small effect size of AS, which may explain the lack of a significant change in AS despite a clear increase in SMR.

The TPC of AS in fish has been suggested to be a bell‐shaped curve (Pörtner & Farrell, 2008), but empirical results vary depending on exposure duration and acclimation conditions. Over short time scales, AS may peak and then decline with further warming: for example, in pink salmon (*O. gorbuscha* Walbaum 1792), AS peaked at 21°C and decreased at higher temperatures, although fish were only exposed for up to 2 days (Clark et al., 2011). Similarly, studies at higher temperatures or with longer acclimations suggest a post‐peak decline in AS. In *S. salar* parr acclimated for 3 weeks, AS decreased from 15 to 21°C (Nuic et al., 2024), suggesting prolonged exposure to heatwaves could lead to aerobic collapse.

In contrast, some species show a continuous increase in AS with rising temperature. For instance, Murray cod (*Maccullochella peelii peelii* Mitchell 1838) exhibited steadily increasing AS across 14–29°C when acclimated for at least 18 h at each step (Clark et al., 2005). Other studies report plateaus in AS rather than declines. After 8 months of acclimation, minnows (*Phoxinus phoxinus* L. 1758) showed no difference in AS across acclimation temperatures (Závorka et al., 2020). Similarly, in round gobies (*Neogobius melanostomus* Pallas 1814) acclimated for 3 weeks, AS increased initially but then plateaued between 15 and 28°C (Christensen et al., 2021). Morissette et al. (2021) found no differences below 23°C in *S. salar*; however, their metabolic rates are measured at a common temperature rather than the acclimation temperature, causing difficult interpretation of their results. Given the diversity of AS responses across studies and the influence of both acclimation duration and temperature exposure, our observation of a plateau in AS at higher temperatures is consistent with previous findings.

In *S. salar* post‐smolts, AS was increased with 4‐week acclimations at temperatures from 3 to 13°C, and plateaued from 13°C up to temperatures of 23°C. Interestingly, fish condition and swim speed dropped and mortality increased (Hvas et al., 2017). This is consistent with widely reported upper thermal limits of *S. salar* at 23°C (Elliott & Elliott, 2010), and reports of optimal temperature for growth of 16°C (Jensen et al., 1989). Our results agree with Hvas et al. (2017) as we found AS plateaued at 16–22°C. The absence of an increase suggests that physiological systems supporting oxygen uptake such as cardiac function, gill function and haemoglobin oxygen affinity are no longer able to scale with increasing thermal pressure (Anttila et al., 2014; Schulte & Healy, 2022). At 16°C *S. salar* may already have been operating near their upper capacity for oxygen uptake and delivery as seen by the lack of significant increase in MMR (Clark et al., 2011; McKenzie & Claireaux, 2010; Norin & Clark, 2016; Sandblom et al., 2016). In contrast, in Sockeye salmon (*O. nerka* Walbaum 1792) and *O*. *kisutch*, MMR continuously increased in acclimation until 22–25°C, where it plateaued (Eliason et al., 2011). This plateau may reflect salmon maximum aerobic capacity, similar to what may be occurring in the present study.

AS is proposed to be of ecological significance because it defines the upper limit for oxygen allocation by fish to sustain aerobic activities such as foraging, digestion, tissue deposition, migration, reproduction and so forth (Claireaux & Lefrançois, 2007; Farrell et al., 2009; Fry, 1971; Pörtner, 2010; Schulte, 2015). For example, reduced appetite could be behaviourally driven to avoid anaerobic metabolism, as suggested by the reduction in feed intake during low AS (review by Jutfelt, 2020). In support of this, reduced growth and lower condition factor at higher temperatures have previously been documented in *S. salar*, when comparing increases to temperatures up to 19°C (Hevrøy et al., 2015; Kullgren et al., 2013). Additionally, in salmon post‐smolts, post‐stress peak cortisol levels significantly increased with higher temperatures (Madaro et al., 2018). This suggests despite stable aerobic capacity to cope with temperature increases, costly physiological mechanisms may be occurring, and therefore a plateau in AS does not necessarily suggest broad optimum temperature range. We can predict that at high temperatures, wild *S. salar* would either seek thermal refuge or compromise aspects of their performance to cope, which could result in poor growth or health (Koskela et al., 1997).

It is important to note both TPCs and upper thermal limits are dependent on acclimation duration and rate of warming (Currie et al., 2014; Lefevre et al., 2021; McKenzie et al., 2021). Most research in fishes has been conducted with stable thermal profiles, whereby each individual experiences only one acclimation temperature (Hvas et al., 2017). In contrast, our study accounts for a cumulative effect of exposure to higher temperatures, using a gradual heating regime. Some acclimation is expected in MMR over short‐term temperature increases (Norin & Clark, 2016; and papers within), which could be a reason for lack of significant difference between groups. Additionally, large variation in MMR and AS could be masking the effect of individual differences in cumulative heat exposure. However, an ecologically relevant scenario for salmon in their environments is a heatwave lasting from a few days to few weeks, and thus these time frames are most relevant to study from a conservation perspective. By testing cumulative effects of a heatwave mirroring wild temperatures, we can better predict how salmon could cope in a realistic exposure to sub‐lethal temperatures (Morash et al., 2018). The general expectation is that longer acclimation with a slower increase of temperature should be beneficial to survival, giving fish time to make compensatory physiological modifications such as displaying plasticity in SMR or MMR, enabling AS to be maintained over a broad range of temperatures. However, in barramundi MMR and AS increased when exposed to acute 10°C warming, due to a higher increase in MMR than SMR, but after 5 weeks of acclimation, AS was similar, mainly due to a reduction in MMR (Norin et al., 2014). A similar pattern was observed in black sea bass (*Centropristis striata* L. 1758) and common triplefin (*Forsterygion lapillum* Hardy 1989), where short‐term exposure caused a raise in AS, before it decreased after some weeks of exposure (Khan et al., 2014; McArley et al., 2017; Slesinger et al., 2019). Consequentially, it could be assumed that longer exposures would further deteriorate aerobic performance.

Although no treatment‐level differences in MMR were detected, individual variation in thermal reaction norms of SMR and MMR may still exist but could not be resolved with our experimental design. This limitation may help explain why AS did not decline despite an increase in SMR, while MMR remained stable on average (Norin et al., 2014; Norin & Metcalfe, 2019; Réveillon et al., 2019). Moreover, sample size was reduced from 128 to 72 fish due to lost data due to computer malfunction (32 points). This resulted in an uneven sample distribution across temperature treatments that may have limited our ability to detect patterns. In addition, one extreme outlier was removed from SMR, MMR and AS analyses based on visual inspection of histograms; although this was a single fish, its exclusion should be acknowledged. Mortality also occurred in respirometers (one, three and two fish at 16, 19 and 22°C, respectively); although this represented <10% of the initial sample size for each group, removing these data introduces some potential for bias. Importantly, our experiment tested only a single hatchery stock, and previous work has shown that fish reared under uniform hatchery conditions would have similar aerobic ceilings (Farrell et al., 2009; Killen et al., 2016). Together, these limitations highlight the need for caution in extrapolating our findings and suggest that broader testing across stocks and larger sample sizes (of individually tracked fish) will be important for future studies.

Recent advances in conservation physiology demonstrate how individual‐level physiological metrics can be used to guide policy and management strategies, as shown in studies on Sockeye salmon in Canada (Patterson et al., 2016). With detailed TPCs, thermal safety margins can be defined in terms of functional success – such as swimming, feeding and reproducing – rather than mortality alone (Pinsky et al., 2019). Temperature‐dependent fisheries management strategies have already been successfully implemented, for example, in Canadian rivers and could be adapted globally to account for population‐specific TPCs (Van Leeuwen et al., 2023). Management strategies, such as adjusting harvest timing and identifying thermal refuges for protection and restoration efforts, could help mitigate impacts of rising temperatures on vulnerable salmon populations.

In recent years, extreme heat events have had catastrophic impacts on migration success and survival across species of salmonids, indicating that the better understanding of their thermal performance is critical for conservation (Baisez et al., 2011; Hinch et al., 2012; Martins et al., 2011, 2012; Muñoz et al., 2015). Our study showed that AS was not increased between 16 and 22°C in juvenile *S. salar* despite a significant increase in SMR. This, combined with increasing metabolic costs of activities, such as swimming and digestion, indicates that stable AS may be leaving juvenile salmon vulnerable to deteriorating performance at temperatures commonly experienced in present‐day heatwaves.

## AUTHOR CONTRIBUTIONS

Lucy Cotgrove and Jenni M. Prokkola: experimental design, conducting experiment, data analysis and writing the manuscript. Jenni M. Prokkola: supervision, management and funding acquisition. Sergey Morozov: equipment and experimental design. Miika Raitakivi and Evan Sala: fish care, equipment maintenance and assistance in data collection.

## FUNDING INFORMATION

This study was funded by the Research Council of Finland (348965 and 353760), Finnish Cultural Foundation (00220693) and Natural Resources Institute Finland.

## CONFLICT OF INTEREST STATEMENT

The authors declare no conflicts of interest.

## Supporting information


Data S1

## Data Availability

Data are hosted on Zenodo data repository (https://doi.org/10.5281/zenodo.15308028), and code for analysis is hosted on Github (https://github.com/jprokkola/Salmon-aerobic-scope-2023-public.git).
